# Relative biological effectiveness of low-energy X-rays (25 kV) in mutant p53 cancer cells

**DOI:** 10.1007/s00411-022-01014-z

**Published:** 2023-01-07

**Authors:** Bettina Habelt, Wolfgang Dörr

**Affiliations:** 1 Department of Radiotherapy and Radiation Oncology, Medical Faculty Carl Gustav Carus, University of Technology Dresden, Dresden, Germany; 2 Present Address: Department of Psychiatry & Psychotherapy, Medical Faculty Carl Gustav Carus, University of Technology Dresden, Dresden, Germany; 3 Department of Radiation Oncology, Medical University Vienna, Vienna, Austria

**Keywords:** RBE, Soft X-rays, Mammography, p53, Breast cancer, Pancreatic cancer

## Abstract

Low-energy X-rays as used in radiation therapy and diagnostics such as mammography are associated with a certain risk of promoting tumour development, especially in patients with mutations in cancer-related genes like TP53. The present study therefore addressed the relative biological effectiveness (RBE) of low-energy X-rays for two human adenocarcinoma cell lines of the breast (MDA-MB-468) and pancreas (BxPC-3) with a mutation in the TP53 gene. Clonogenic survival and cytogenetic changes in terms of micronuclei (MN) formation were determined following irradiation with 25 kV X-rays and 200 kV reference irradiation in the dose range of 1–8 Gy. Except the frequency of MN-containing binucleated cells (BNC) (BNC + MN/BNC) in breast cancer cells yielding an RBE between 0.6 and 0.8, both cell lines displayed dose-dependent variations of RBE values between 1 and 2 for all biological end points (cell survival, (BNC + MN/BNC), MN/BNC, MN/(BNC + MN)) with increased effectiveness of 25 kV irradiation in pancreatic compared to breast cancer cells. The results confirm previous findings indicating increased effectiveness of low-energy X-rays and underline the necessity of careful risk estimation for cancer screening programmes.

## Introduction

Breast cancer is the most common malignant tumour and the leading cause of cancer death in women worldwide (Bray et al. 2018). Regular mammography screening for early detection is recommended for women aged 50 to 74 years (Siu & on behalf of the U.S. Preventive Services Task Force 2016). However, mammography involves application of low-energy X-rays of about 25–35 kV which are associated with a certain risk of promoting tumour development (Pauwels et al. 2015). Therefore, precise determination of the relative biological effectiveness (RBE) of X-rays in this energy range is mandatory. The International Commission on Radiological Protection recommends a weighting factor *w*_*R*_ = 1 for photons of all energies (ICRP 2007). In contrast, in vitro studies suggest that the RBE varies depending on photon energy, cell line and biological end point (Paget et al. 2019), with low-energy X-rays being more effective per unit dose than photons of higher energies (Beyreuther et al. 2012; Heyes et al. 2006). X-ray irradiation of non-tumourigenic breast epithelial cell lines revealed RBE values in MCF-12A cells for clonogenic survival and micronuclei formation of 1.1–1.4 at 25 kV (Lehnert et al. 2006) and 1.2–4.1 at 10 kV relative to 200 kV photons (Lehnert et al. 2008). For chromosomal aberrations including acentric fragments, dicentric chromosomes and centric rings, similar radiation treatment of MCF-12A and 184A1 cells resulted in an RBE of 0.97 ± 0.10 and 1.31 ± 0.21 (25 kV) and 1.92 ± 0.26 and 1.40 ± 0.12 (10 kV) (Beyreuther et al. 2009). Likewise, FISH-based examination of complex chromosomal damages in terms of translocations and dicentrics in 184A1 cells revealed increasing frequency and complexity of chromosomal aberrations with decreasing X-ray energy associated with RBE values of 0.84 ± 0.09 at 25 kV and 1.22 ± 0.18 at 10 kV. The minimum number of DNA strand breaks to build a visible chromosomal damage resulted in similar RBE ratios of 0.93 ± 0.07 (25 kV) and 1.25 ± 0.10 (10 kV) (Beyreuther et al. 2012). In MCF-10A cells receiving a single mammogram, Mills et al., (2015) calculated an RBE of 29 kV X-rays relative to ^137^Cs radiation of 1.1 ± 0.2 for DNA double-strand breaks based on 53BP1 foci assay representing no significant difference between the radiation qualities. However, repeated mammograms increased the occurrence of 53BP1 foci.

Though there is evidence for reduced breast cancer mortality due to organised screening programmes (Zielonke et al. 2020), increased risk of cancer due to diagnostic X-rays (Heyes et al. 2006; Hong et al. 2019) and a potential influence on growth and metastasis of latent tumours should not be underestimated. The present study therefore addressed the RBE of low-energy X-rays for clonogenic survival and chromosomal damage in breast adenocarcinoma cells (MDA-MB-468). We further included BxPC-3 pancreatic cancer cells in our study. Pancreatic cancer has one of the highest cancer mortality rates as it is mostly detected at an advanced, incurable stage. Therefore, early detection through screening of individuals belonging to high-risk groups is recommended also for this type of cancer (Pereira et al. 2020). High risk is based, for example, on family history of the disease and a genetic susceptibility. Both cell lines used in the present study carry a mutation in the tumour suppressor gene *TP53*.

*TP53* codes for the transcription factor p53 and is by far the most frequently mutated gene in the majority of human cancers, including breast cancer and pancreatic cancer (Cicenas et al. 2017; Duffy et al. 2018). P53 is crucial for DNA repair and tumour development. It is activated in response to mutagenic and cytotoxic stressors, such as ionising radiation. Enhanced p53 expression due to DNA damages, such as single- and double-strand breaks, interrupts the cell cycle to enable repair or induces apoptosis depending on the extent of damage (Menon & Povirk 2014). Mutations in p53 disable normal p53 tumour suppressor function by blocking sequence-specific DNA-binding activity. Disturbed apoptotic mechanisms consequently enhance cell proliferation and metastasis of cancer cells and also alter their response to radiotherapy (Kong et al. 2021).

## Materials and methods

### Cell culture

The cell line BxPC-3 was established from an adenocarcinoma of the pancreas of a 61-year-old female (Tan et al. 1986). It carries a G-A-mutation in codon 220 of p53 causing a tyrosine/cysteine substitution. The cell line MDA-MB-468 was isolated from a pleural effusion of a 51-year-old woman with metastatic adenocarcinoma of the mamma (Cailleau et al. 1978). It contains a G-A-mutation in codon 273 of p53 resulting in an arginine/histidine substitution.

Both cell lines were grown in T25-cell culture flasks (Nunc, Wiesbaden, Germany) with 5 ml of culture medium. BxPC-3 cells were cultured in RPMI-1640 medium containing 10% FCS, 1% sodium bicarbonate (100 mM), 1% HEPES buffer (1 mM), 1% penicillin/streptomycin (1 mg/ml) and 0.5% glucose (45%) at 37 °C in 5% carbon dioxide. MDA-MB-468 cells were seeded in Leibowitz L-15 medium containing 10% FCS, 1% penicillin/streptomycin (1 mg/ml) and 1% glutamine (200 mM) at 37 °C without carbon dioxide fumigation. Media was changed every 2 days. Cells were subcultured before reaching 70% confluence.

### Irradiation

The tungsten anode X-ray tube Darpaq 150-MC (Raytech Ltd. Swindon, UK, operated at 25 kV, 20 mA, 0.25 mm aluminium filter, dose rate 1.954 Gy/min) was used for low-energy irradiation. To minimise the attenuation of soft X-rays in the medium, T25 cell culture flasks were placed upside down in a polystyrene holder underneath the X-ray tube. As such, cells were not covered with medium during irradiation.

The reference irradiation was performed with a Yxlon Maxishot X-ray tube (Yxlon International X-Ray GmbH, Copenhagen, Denmark, operated at 200 kV, 20 mA, 0.5 mm copper filter, dose rate 1.317 Gy/min). The cells remained in culture flasks covered with medium and were placed in a horizontal holder within the vertical beam path. In case of both, 25 kV and 200 kV, cells were irradiated with 0, 1, 2, 3, 4, 5, 6, 7 or 8 Gy.

### Clonogenic assay

To determine clonogenic survival, each of two Petri dishes with three different cell concentrations per dose were seeded immediately following irradiation. With increasing irradiation dose, increasing cell concentrations were chosen to get comparable colony numbers. This had to be tested individually for each cell line. The duration of subsequent incubation was dependent on the cell line and had to be assessed by daily checking the colony number under a microscope with the aim of getting many, but separate colonies. The colony forming assay was stopped after incubation for 10 (BxPC-3) and 12 (MDA-MB-468) days, respectively, by removing the media and washing the Petri dishes twice with 3 ml PBS. Then cells were fixed with 5 ml of 80% ethanol for 15 min. Following removal of ethanol, cells were washed with tap water and stained for 10 min with 5 ml crystal violet (Sigma Aldrich) per dish. The Petri dishes were washed several times with tap water and dried in air.

Counting of colonies, defined by a cell number > 50 cells, was done with a light microscope at 2.5 × magnification. Plating efficiency (PE) was calculated by dividing the number of counted colonies with the seeded amount of cells. The surviving fraction (SF) was derived from the PE at a specified irradiation dose divided by the PE of non-irradiated cells.

### Micronucleus assay

The formation of micronuclei (MN) had to be assessed within cells that underwent exactly one division and appear as binucleate cells which is achieved by adding the mycotoxin cytochalasin B (Sigma Aldrich) to the medium, which blocks cytokinesis, but not karyokinesis (Fenech 1993). Cell line-dependent cytochalasin B concentrations and subsequent incubation times to achieve many binucleate cells were evaluated in advance yielding cytochalasin B concentrations of 2 µg/ml (BxPC-3) and 1.5 µg/ml (MDA-MB-468) and an incubation time of 48 h for both cell lines.

For each cell line and radiation quality (25 kV, 200 kV), the micronuclei assay was performed three to four (BxPC-3, 200 kV only) times. Thereby, each assay included a repeat determination for each of the applied doses between 0 and 8 Gy. Following irradiation, 50,000 cells each were seeded in 21 cm^2^ Petri dishes (Nunc) and incubated for 24 h. The media was replaced by media containing cytochalasin B in the previously determined concentrations followed by 48 h incubation. After removal of media, cells were washed for 5 min with 5 ml of 0.9% NaCl and 5 ml methanol (− 20 ℃) for 10 min before staining with 5% Giemsa solution (5 ml per dish) for 15 min. Finally, dishes were washed several times with tap water and dried overnight. Cells were examined under a light microscope at 200 × magnification. Per dish, 200 binucleated cells (BNC) were determined and amongst them the fraction of binucleated cells containing micronuclei ((BNC + MN)/BNC), the number of micronuclei per binucleated cell (MN/BNC) and per micronuclei-containing binucleated cell (MN/(BNC + MN)). Micronuclei were identified according to the criteria of Fenech (1993).

### Data evaluation

Statistical data evaluation was performed using the Statistical Analysis System (SAS), Version 9.1.3. Dose dependency of clonogenic survival for each cell line based on mean values for PE and SF of each radiation quality and dose was analysed using the linear quadratic (LQ) function $\log SF = \alpha_{SF} \cdot D + \beta_{SF} \cdot D^{2}$ with survival fraction SF at dose D. The RBE was determined for the initial slope α_SF_ of the survival curves and for the clonogenic survival of 10%, 50% and 90%. In addition, we compared clonogenic survival at 2 Gy (SF_2_).

For the parameters derived from the micronucleus assay, i.e. BNC, (BNC + MN)/BNC, MN/BNC and MN/(BNC + MN), mean values were calculated for each dose and radiation quality and fitted using an LQ model y = ax^2 ^+ bx + c and nonlinear regression. The RBE was determined as the ratio of the isoeffective doses of 200 kV and 25 kV radiation (RBE = D_200kV_/D_25kV_).

## Results

### Cell survival

**Fig. 1:**
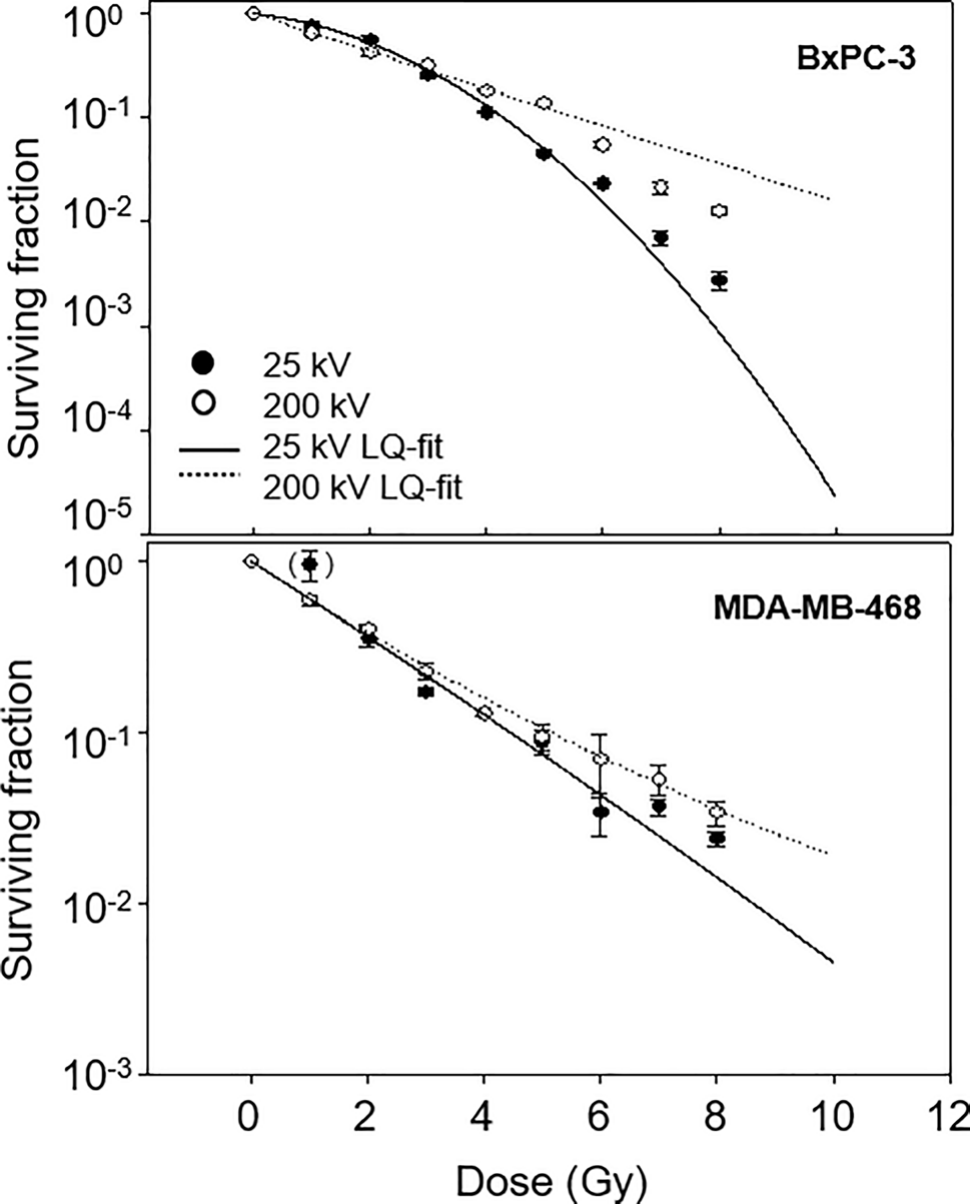
Survival of BxPC-3 (top) and MDA-MB-468 cells (bottom) after irradiation with 25 kV X-rays. (black circles, solid line) compared to 200 kV X-rays (white circles, dashed line) as mean ± SEM from three to four experiments

The clonogenic survival data following irradiation with 25 kV and 200 kV X-rays are presented in Table 1 and Fig. 1 as means with standard errors (SEM) and standard deviation (SD). Plating efficiency of BxPC-3 cells was 8 – 10%, for MDA-MB-468 cells < 1%.

**Table 1:** Surviving fraction (SF) with SEM and SD of cell lines BxPC-3 and MDA-MB-468 following irradiation with 25 kV and 200 kV X-rays

	BxPC-3						MDA-MB-468					
	25 kV			200 kV			25 kV			200 kV		
Dose (Gy)	SF	SEM	SD	SF	SEM	SD	SF	SEM	SD	SF	SEM	SD
0	1.000	0.000	0.000	1.000	0.203	0.406	1.000	0.000	0.000	1.000	0.000	0.000
1	0.772	0.062	0.107	0.654	0.159	0.318	0.965	0.198	0.343	0.596	0.045	0.078
2	0.565	0.039	0.068	0.409	0.084	0.168	0.359	0.040	0.069	0.404	0.020	0.035
3	0.257	0.016	0.028	0.356	0.031	0.062	0.173	0.010	0.017	0.228	0.025	0.043
4	0.113	0.005	0.009	0.183	0.018	0.036	0.131	0.005	0.009	0.131	0.006	0.010
5	0.046	0.003	0.005	0.141	0.011	0.022	0.089	0.014	0.024	0.095	0.017	0.029
6	0.023	0.001	0.002	0.055	0.007	0.014	0.034	0.010	0.017	0.070	0.028	0.048
7	0.007	0.001	0.002	0.022	0.005	0.010	0.037	0.004	0.007	0.054	0.011	0.019
8	0.002	0.001	0.002	0.013	0.002	0.004	0.024	0.002	0.003	0.034	0.005	0.009

Initially, survival of BxPC-3 cells decreased earlier after 200 kV irradiation than after 25 kV. At higher doses ≥ 4 Gy, cell numbers decreased steeply following 25 kV irradiation compared to a much shallower behaviour at 200 kV with higher surviving fractions.

MDA-MB-468 survival decreased linearly after 25 kV irradiation. A similar behaviour was initially observed at 200 kV. At higher doses ≥ 4 Gy, cell survival followed a slightly concave curve shape associated with higher surviving fractions compared to 25 kV irradiation.

The linear quadratic analysis of the data showed a significant fitting of both curves (*p* < 0.0001). Coefficients of the LQ model and corresponding RBE values are displayed in Table 2.

**Table 2:** Coefficients ± standard error (SE) of the linear quadratic model for the survival of cell lines BxPC-3 and MDA-MB-468 after irradiation with 25 kV and 200 kV X-rays together with the survival rate at 2 Gy (SF_2_) and with the RBE for the initial slope (RBE_α_) and RBEs at the 90% (RBE_0.9_), 50% (RBE_0.5_) and 10% (RBE_0.1_) survival level

Cell line	Radiation quality	α ± SE	β ± SE	SF_2_ ± SE	RBE_α_	RBE_0.9_	RBE_0.5_	RBE_0.1_
BxPC-3	25 kV	0.417 ± 0.042	− 0.003 ± 0.019	0.565 ± 0.039	2.6	0.5	0.8	1.3
	200 kV	0.137 ± 0.046	0.093 ± 0.025	0.399 ± 0.026				
MDA-MB-468	25 kV	0.498 ± 0.028	0.004 ± 0.011	0.359 ± 0.041	1.0	1.0	1.0	1.0
	200 kV	0.499 ± 0.031	− 0.010 ± 0.011	0.404 ± 0.021				

For BxPC-3 cells, α-coefficients of 0.14 and 0.42 for 200 kV and 25 kV irradiation were determined. An RBE of 0.3 for the initial slope of the curve, α_25_/α_200_, was calculated. With values of ~ 0 for 25 kV, β-coefficients differed significantly corresponding to an absent bending of the curve. Surviving fractions of 0.57 (25 kV) and 0.4 (200 kV) at 2 Gy correspond to a less effective reference irradiation with a factor of 0.8. RBE values for cell survival of 90%, 50% and 10% were 0.5, 0.8 and 1.3.

Cell survival analysis of MDA-MB-468 resulted in identical α-coefficients of 0.5 for both radiation qualities; β-coefficients were not significantly different from 0 corresponding to a linear curve shape for both, 25 kV and 200 kV X-rays. Also, SF_2_ values were similar for both radiation qualities resulting in an RBE of 1.0 for all parameters.

### Micronuclei formation

#### Incidence of binucleated cells in dependence of radiation dose

**Fig. 2:**
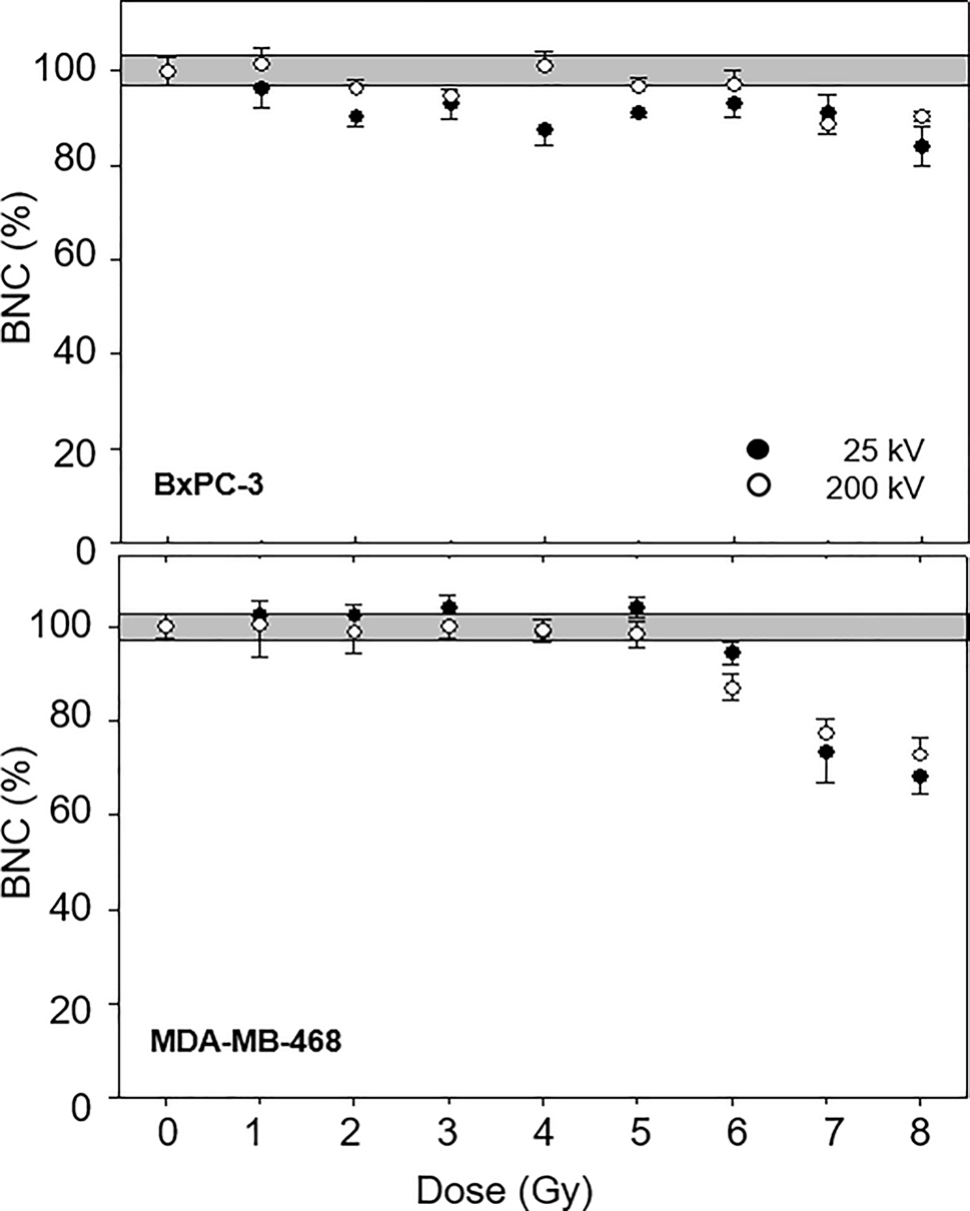
Proportion of binucleated cells within the total cell population of BxPC-3 (top) and MDA-MB-468 (bottom) in dependence of radiation dose for 25 kV X-rays (black circles) compared to 200 kV X-rays (white circles) as mean ± SEM

Dose-dependent fractions of BNC within the cell populations are illustrated in Fig. 2. Following 200 kV irradiation up to 6 Gy, BxPC-3 cells displayed similar numbers of BNC compared to non-irradiation. At higher doses, a progressive decrease of the fraction of BNC due to an increasing proliferation inability was observed. After 25 kV irradiation, decreased BNC fractions were already observable from 2 Gy onwards. For both radiation qualities, the frequency of of BNC in MDA-MB-468 cell populations corresponded to non-irradiated conditions up to a dose of 5 Gy, followed by a progressive decrease of BNC for both, 25 kV and 200 kV irradiation.

#### Frequency of binucleated cells with micronuclei

**Fig. 3:**
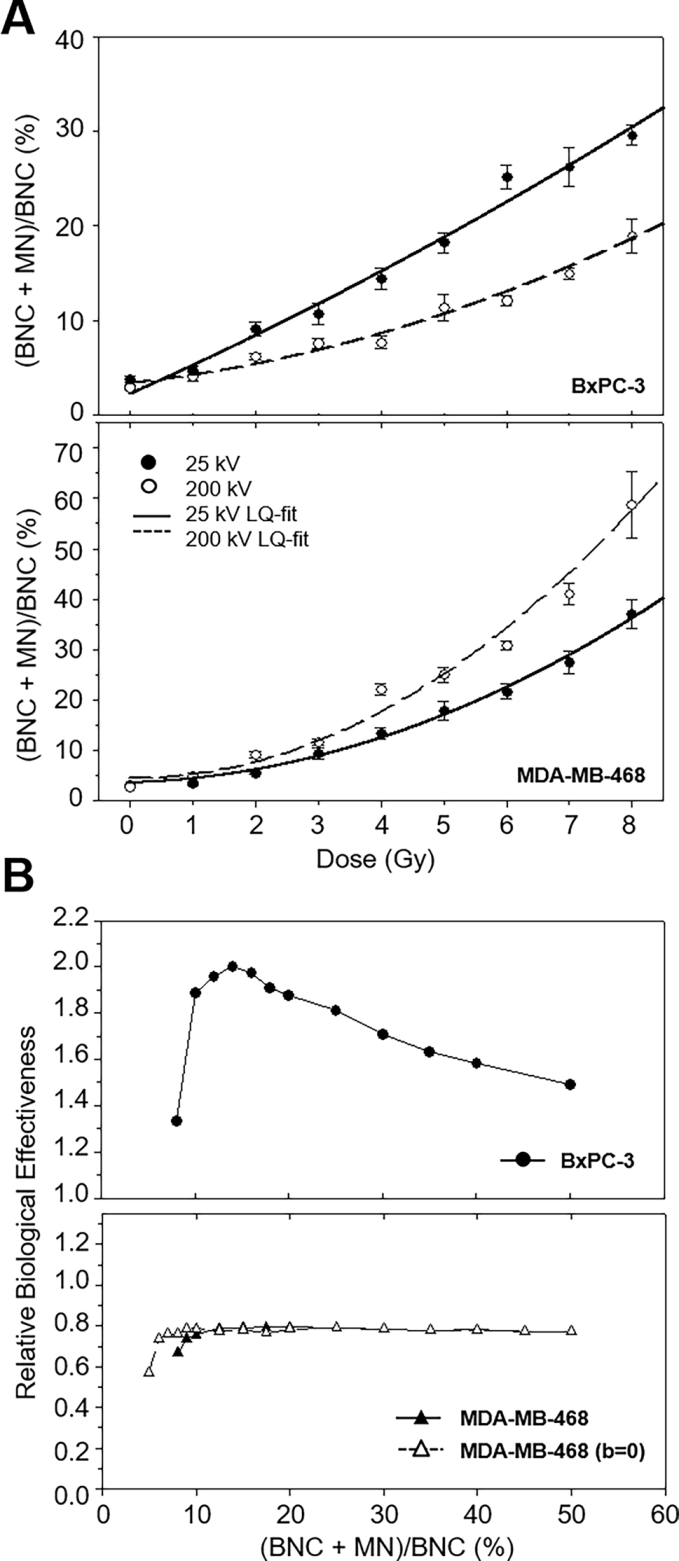
**A** Proportion of binucleated cells carrying micronuclei within the population of binucleated cells of BxPC-3 (top) and MDA-MB-468 (bottom) in dependence of radiation dose for 25 kV X-rays (black circles) compared to 200 kV X-rays (white circles) as mean ± SEM. The curve fit represents the parameters summarised in Table 3. **B** RBE for the fraction of micronuclei-containing binucleated cells of BxPC-3 (top) and MDA-MB-468 (bottom). For the breast cancer cell line, the analysis was repeated for the adjusted curve with b = 0 (white triangles) as the original curve had a negative term b (black triangles)

Dose-dependent numbers of MN-containing BNC are illustrated in Table 3 and Fig. 3A. Both cell lines and radiation qualities displayed a progressive increase of the population of BNC with MN in a linear quadratic dose–effect relationship with *p* < 0.0001. In BxPC-3 cells, dose–response following 200 kV irradiation resulted in a convex curve shape, while a linear increase of the fraction of MN-containing BNC was observed following 25 kV. At all doses, (BNC + MN)/BNC following low-energy irradiation exceeded those after 200 kV irradiation reflecting a higher effectiveness at similar doses. Due to the different curve shapes, the RBE depended on the analysed effect level (Fig. 3B). RBE values increased with dose and were similar at 1 Gy. The portion of MN in 5% and 10% of BNC resulted in an RBE of 1.9, and for larger numbers, RBE values decreased to 1.5. The RBE for the initial slope of the curve, b_25_/b_200_, equaled 4.1.

**Table 3:** Coefficients ± SE of the linear quadratic model for the fraction of binucleated cells with micronuclei (BNC + MN/BNC) of cell lines BxPC-3 and MDA-MB-468 after irradiation with 25 kV and 200 kV X-rays

Cell line	Radiation quality	a ± SE	b ± SE	c ± SE	*p*
BxPC-3	25 kV	0.0716 ± 0.0972	2.9526 ± 0.8934	2.2539 ± 1.8283	< 0.0001
	200 kV	0.1487 ± 0.0454	0.7259 ± 0.4350	3.3550 ± 0.9384	< 0.0001
MDA-MB-468	25 kV	0.4616 ± 0.0789	0.3936 ± 0.7967	3.5939 ± 1.8818	< 0.0001
	200 kV	0.9569 ± 0.2155	− 1.3297 ± 2.2371	7.2642 ± 5.1609	< 0.0001
	200 kV^a^	0.8312 ± 0.0399^a^	0.0000 ± 0.0000^a^	4.4590 ± 1.9899^a^	< 0.0001^a^

^a^Analysis performed with b = 0

In contrast, 200 kV reference irradiation induced larger fractions of MN-containing BNC than 25 kV at all doses in MDA-MB-468 cells with RBE values  < 1 throughout the whole effect range reflecting a reduced effectiveness of low-energy radiation compared to the reference radiation. At a low effect level of 5% BNC with MN, RBE is about 0.6 and stabilised at 0.8 at ≥ 10%. Due to the negative b-coefficient, the initial slope for 200 kV was not assessed.

#### Number of micronuclei per binucleated cell

**Fig. 4:**
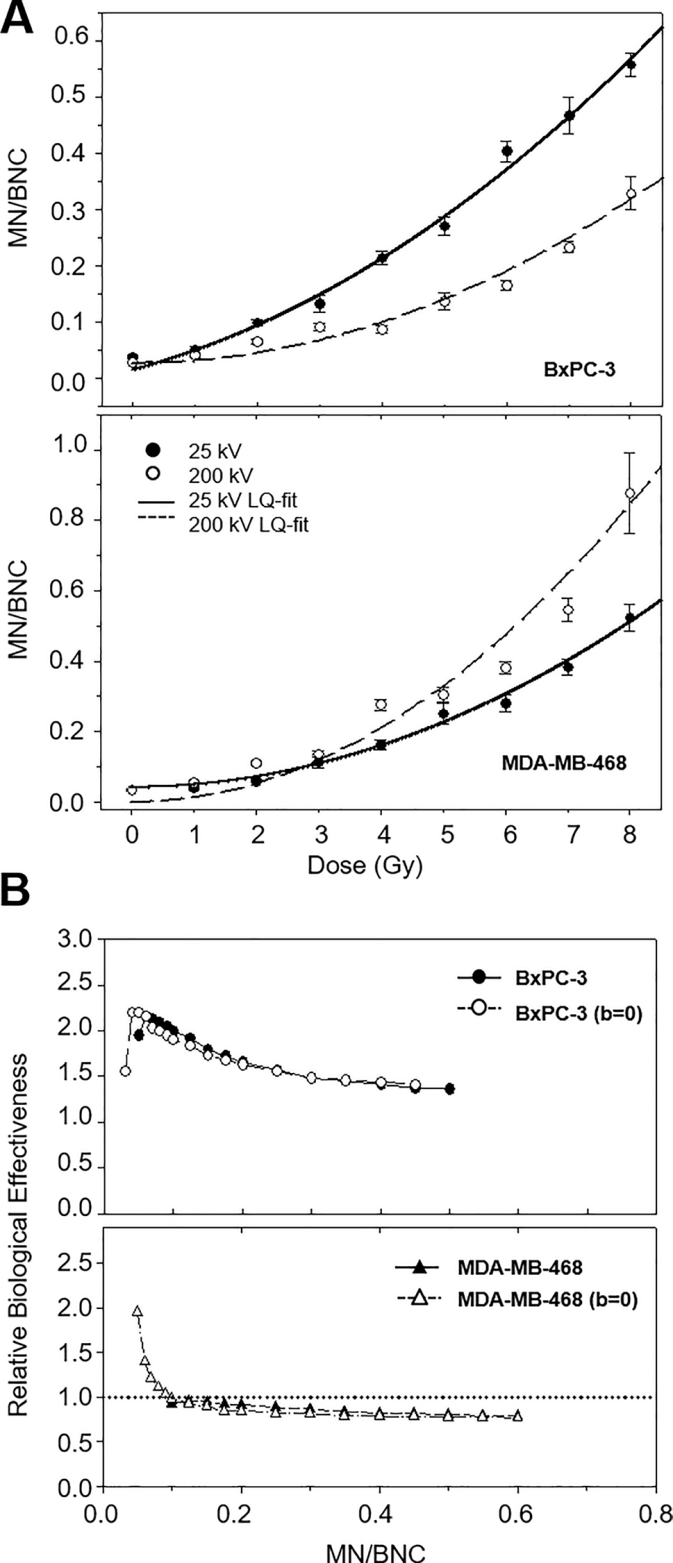
**A** Frequency of micronuclei per binucleated cell of BxPC-3 (top) and MDA-MB-468 (bottom) in dependence of radiation dose for 25 kV X-rays (black circles) compared to 200 kV X-rays (white circles) as mean ± SEM. The curve fit represents the parameters of Table 4 with adjusted values for 200 kV (b = 0). **B** RBE for the frequency of micronuclei per binucleated cell of BxPC-3 (top) and MDA-MB-468 (bottom). For both cell lines, the analysis was repeated for the adjusted curves with b = 0 (white symbols) due to the negative term b of the original curves (black symbols)

The average frequency of micronuclei per binucleated cell (MN/BNC) followed a linear quadratic dose–response relationship as well with *p* < 0.0001 (Table 4). For 200 kV irradiation, linear quadratic analyses were repeated with b = 0 due to a negative b-coefficient not significantly different from zero (Fig. 4A).

**Table 4:** Coefficients ± SE of the linear quadratic model for the number of micronuclei per binucleated cell (MN/BNC) of cell lines BxPC-3 and MDA-MB-468 after irradiation with 25 kV and 200 kV X-rays

Cell lines	Radiation quality	a ± SE	b ± SE	c ± SE	*p*
BxPC-3	25 kV	0.0049 ± 0.0013	0.0301 ± 0.0124	0.0156 ± 0.0266	< 0.0001
	200 kV	0.0059 ± 0.0009	− 0.0142 ± 0.0092	0.0550 ± 0.0203	< 0.0001
	200 kV^a^	0.0045 ± 0.0003^a^	0.0000 ± 0.0000^a^	0.0275 ± 0.0104^a^	< 0.0001^a^
MDA-MB-468	25 kV	0.0073 ± 0.0015	0.0004 ± 0.0155	0.0423 ± 0.0369	< 0.0001
	200 kV	0.0210 ± 0.0048	− 0.0817 ± 0.0504	0.1720 ± 0.1208	< 0.0001
	200 kV^a^	0.0132 ± 0.0007^a^	0.0000 ± 0.0000^a^	0.0000 ± 0.0000^a^	< 0.0001^a^

^a^Analysis performed with b = 0

While at 1 Gy both radiation qualities induced comparable levels of MN/BNC, the cell lines differed in their response to higher doses: in contrast to MDA-MB-468 cells, low-energy irradiation generated larger numbers of MN/BNC than the reference irradiation in BxPC-3 cells, reflecting a higher biological effectiveness. The corresponding RBE values are presented in Fig. 4B. In BxPC-3 cells, the RBE was initially about ~ 2, decreasing progressively to ~ 1.4 with increasing MN incidence. In MDA-MB-468 cells, RBE initially equaled 2 and decreased to ~ 0.8.

#### Number of micronuclei per MN-containing binucleated cell

**Fig. 5:**
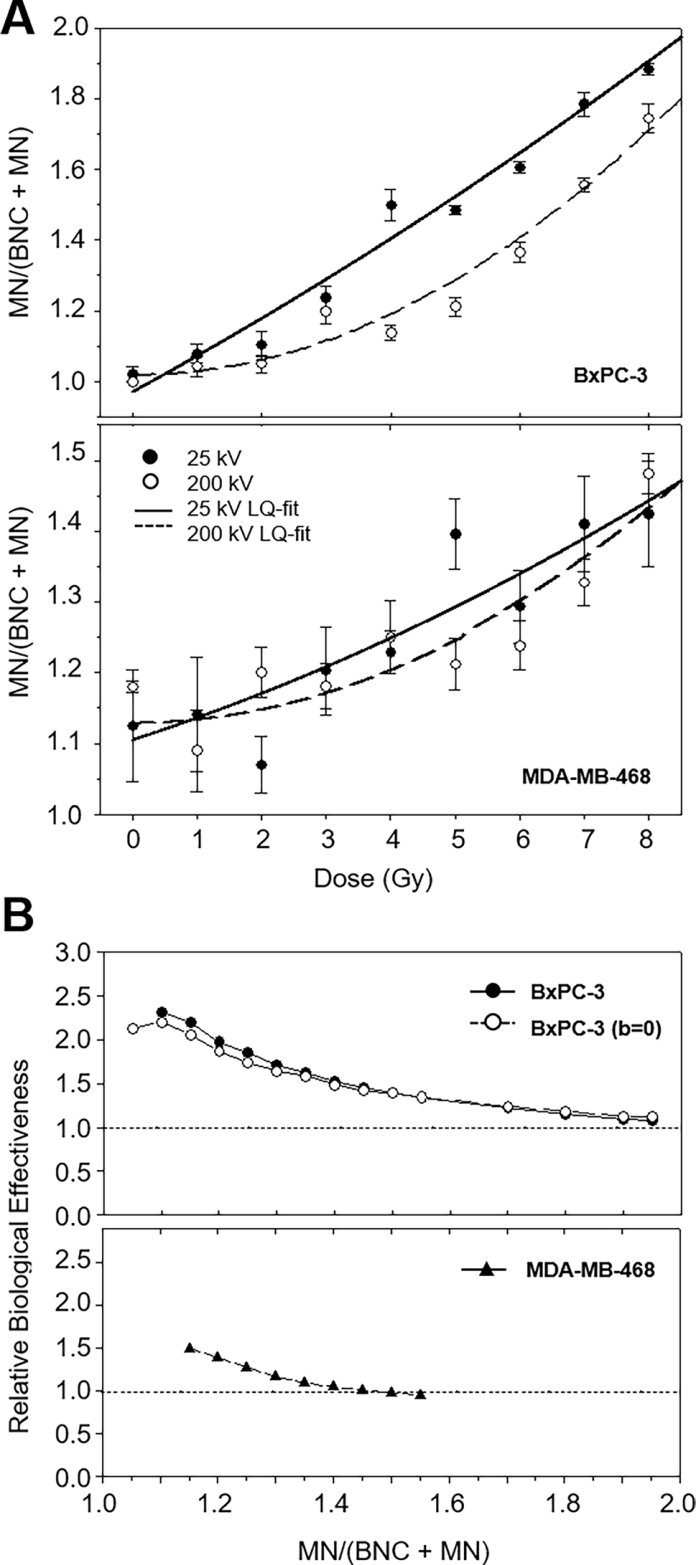
**A** Frequency of micronuclei per micronuclei-containing binucleated cell of BxPC-3 (top) and MDA-MB-468 (bottom) in dependence of radiation dose for 25 kV X-rays (black circles) compared to 200 kV X-rays (white circles) as mean ± SEM. The curve fit represents the parameters of Table 5. **B** RBE for the frequency of micronuclei per micronuclei-containing binucleated cell of BxPC-3 (top) and MDA-MB-468 (bottom). For the pancreas cancer cell line, the analysis was repeated for the adjusted curves with b = 0 (white circles) due to the negative term b of the original curve (black circles)

The frequency of micronuclei per MN-containing binucleated cell (MN/(BNC + MN)) is presented in Fig. 5A.

In BxPC-3 cells, 25 kV induced more MN than 200 kV irradiation at all doses, while radiation response of MDA-MB-468 cells displayed pronounced variations. In both cell lines, irradiation with 25 kV displayed in a linear curve shape, while a linear quadratic response was observed following 200 kV irradiation (Table 5). The different curve shapes cause a varying RBE depending on the effect level (Fig. 5B). In BxPC-3 cells, initial RBE values of 2–2.5 approached progressively to 1 with increasing radiation effect. A similar behaviour was observed for MDA-MB-468 cells with an initial RBE of 1.5 and a steeper decline.

**Table 5:** Coefficients ± SE of the linear quadratic model for the number of micronuclei per micronuclei-containing binucleated cell (MN/BNC + MN) of cell lines BxPC-3 and MDA-MB-468 after irradiation with 25 kV and 200 kV X-rays

Cell line	Radiation quality	a ± SE	b ± SE	c ± SE	*p*
BxPC-3	25 kV	0.0021 ± 0.0043	0.0996 ± 0.0357	0.9712 ± 0.0617	< 0.0001
	200 kV	0.0154 ± 0.0042	− 0.0436 ± 0.0401	1.0979 ± 0.0794	0.0002
	200 kV^a^	0.0108 ± 0.0009^a^	0.0000 ± 0.0000^a^	1.0185 ± 0.0317^a^	< 0.0001^a^
MDA-MB-468	25 kV	0.0016 ± 0.0032	0.0298 ± 0.0263	1.1047 ± 0.0410	0.0023
	200 kV	0.0046 ± 0.0036	0.0003 ± 0.0308	1.1286 ± 0.0555	0.0059

^a^Analysis performed with b = 0

## Discussion

In the present study, the dose–response relationships for clonogenic cell survival and cytogenetic changes in terms of micronuclei formation following irradiation with low-energy 25 kV X-rays relative to 200 kV were examined in p53-mutated cancer cells of the breast and pancreas. The dose–effect curves were fitted with the linear quadratic model as the established method of cellular radiation effects.

The majority of studies focusing on the RBE of low-energy X-rays of 25–30 kV, as used for mammography, yielded values between 1 and 2 (for review see Mills et al. (2015)). Also, the results of the present study are in this range. For clonogenic survival, the reference irradiation resulted in an almost linear dose–effect curve in BxPC-3 cells, while 25 kV irradiation displayed a typical shoulder curve with RBE values of  < 1 at low doses and  > 1 at doses above 3 Gy. For MDA-MB-468 cells, dose–effect curves of both, 25 kV and 200 kV, were linear and almost identical resulting in an RBE = 1. In both cell lines, analysis of micronuclei formation showed decreasing levels of BNC at higher doses > 6 Gy due to limited proliferation ability. The fraction of MN-containing BNC of BxPC-3 reflected a higher effectiveness of 25 kV with an RBE of 1.4–1.9. In contrast, there was a decreased effectiveness of the low-energy irradiation for MDA-MB-468 associated with RBE values of 0.6–0.8. For the number of MN per BNC, RBE values ranged between 1.4 and 2.2 for BxPC-3 cells, whereas for MDA-MB-468 the reference irradiation showed a smaller effectiveness (decreasing RBE values between 2 and 1) at lower doses < 3 Gy, while it was more effective at higher doses with an RBE ~ 0.8. For the number of MN per MN-containing BNC, both cell lines displayed a linear quadratic dose–effect relationship for 200 kV and a linear progression following 25 kV X-rays. The resulting RBE values decreased progressively down to 1 from 2.3 (BxPC-3) and 1.5 (MDA-MB-468), respectively. In summary, 25 kV X-rays were more effective compared to 200 kV reference irradiation for all end points in pancreatic cancer cells different from breast cancer cells. Based on the reduced sensitivity, we found for MDA-MB-468 cells towards 25 kV radiation, the influence of further decreasing the energy of diagnostic X-rays might be worth testing to further reduce the risk of regular mammography screenings.

The influence of p53 status on radiosensitivity has mostly been examined in the context of radiotherapy that uses high-energy gamma radiation. Thereby, mutant p53 and p53^null^ status have been associated with both, increased and decreased radiosensitivity (Kong et al. 2021).

These studies used a variety of cell lines such as mouse fibroblasts, rat lung epithelial cells and different human cancer lines and often compared different cell lines with and without p53 mutations. However, to what extent radiation responses are based on the characteristics of the cell lines or are related to the p53 status needs to be determined within the same cell line with vs. without a p53 mutation, e.g. through transfection of intact wild-type p53 into mutant cells. This has unfortunately also not been done here and therefore requires additional investigations enabling direct conclusions on the impact of a p53 mutation on sensitivity towards low-energy X-rays.

The present study confirms that the biological effectiveness for different end points varies in dependence of dose. To induce evident effects, cell culture studies are performed with higher doses than used in X-ray diagnostics (Metaxas et al. 2019), making it hardly possible to draw direct conclusions on the effectiveness of low doses as applied in mammography. Thus important in this context, Li et al. (2017a) demonstrated that in p53-mutant MDA-MB-231 but not normal breast cells, low doses of 50–200 mGy X-rays at 12.5 kV accelerated cell growth, induced an accumulation of mutant p53 and upregulated the expression of cyclin-dependent kinases CDK4, CDK6 and cyclin D1, which are involved in the development and survival of numerous cancer types (Goel et al. 2022). Introduction of wild-type p53 into MDA-MB-231 cells abolished irradiation-induced cell proliferation (Li et al. 2017a). Similar radiation treatment inhibited cell growth of p53^null^ type PC-3 prostate cancer cells, but not of the normal prostate cell line RWPE-1. Further, the radiation-induced expression of ataxia-telangiectasia mutated (ATM) protein, observed in both cell lines, only activated the ATM̸p21 pathway and upregulated p21 expression in PC-3, but not in RWPE-1 cells (Li et al. 2017b). ATM kinase plays a critical role in radiation-induced DNA damage response. It activates p21 to induce cell cycle arrest via direct binding to p53 (Mezzomo et al. 2020), though alternative, p53-independent, p21-expression has also been observed (Macleod et al. 1995). Blocking of wild-type p53 in RWPE-1 cells enabled activation of the ATM/p21 pathway and upregulation of p21 in response to radiation (Li et al. 2017b). Stankevicins et al. (2013) demonstrated that low-energy X-rays (5 Gy, 30 kV) are able to increase the expression of miR-34a in normal breast cells MCF-10A and p53-wild-type breast cancer cells MCF-7, while its level remained unchanged in p53-mutant breast cancer cells T-47D. MiR-34a belongs to a family of microRNAs that are activated through p53 and in turn are involved in the expression of various p53-induced genes (Navarro & Lieberman 2015). These findings support the hypothesis that the p53 status differentiates the radiation responses of healthy and tumorous cells which should be taken into account for risk estimation for cancer screening programmes but have also motivated p53-targeted cancer therapies. Small-molecule compounds such as inhibitors of heat shock protein 90 and histone deacetylase aim at reducing tumour growth through depletion of mutant p53, while treatment with e.g. PRIMA-1 (2,2-bis (hydroxymethyl)-1-azabicyclo[2,2,2]octan-3-one) reconstitutes wild-type p53 (Parrales & Iwakuma 2015). These agents have further been shown to increase p53-dependent radiosensitivity in cancer cells (Kim et al. 2010; Perdrix et al. 2017; Shintani et al. 2006). Though such radiosensitisers could increase the effectiveness of radiotherapy and improve local control of radio-resistant tumours, their impact on the risk for recurrence and second tumours due to surveillance screenings with diagnostic low-energy X-rays still needs to be explored.

## Data Availability

The datasets generated during and/or analysed during the current study are available from the corresponding author on reasonable request.
